# Utility of a new notation to visualize flow cytometry analysis results: first preliminary comparison with immunohistochemistry to detect CD30 expression on T-cell lymphoma cells

**DOI:** 10.1186/s12885-021-09098-4

**Published:** 2021-12-20

**Authors:** Fumiyoshi Fujishima, Noriko Fukuhara, Hiroki Katsushima, Yasuhiro Nakamura, Hideo Harigae, Hironobu Sasano, Ryo Ichinohasama

**Affiliations:** 1 Department of Pathology, Tohoku University Hospital, 1-1 Seiryo-machi, Aoba-ku, Sendai, 980-8574 Japan; 2 Department of Hematology and Rheumatology, Tohoku University Graduate School of Medicine, 1-1 Seiryo-machi, Aoba-ku, Sendai, 980-8574 Japan; 3 Department of Diagnostic Pathology, Osaka Red Cross Hospital, 5-30 Fudegasakicho, Tennoji-ku, Osaka, 543-8555 Japan; 4 Division of Pathology, Tohoku Medical and Pharmaceutical University, 4-4-1 Komatsushima, Aoba-ku, Sendai, 981-8558 Japan; 5 Department of Pathology, Tohoku University Graduate School of Medicine, 2-1 Seiryo-machi, Aoba-ku, Sendai, 980-8575 Japan; 6 Division of Hematopathology, Tohoku University Hospital, 1-1 Seiryo-machi, Aoba-ku, Sendai, 980-8574 Japan

**Keywords:** Flow cytometry, CD30, Immunohistochemistry, Malignant lymphoma, Notation

## Abstract

**Background:**

It is important to confirm CD30 expression in T-cell lymphoma cases, but immunohistochemical staining for CD30 is not commonly performed and no comparison has been done between the results of flow cytometry (FCM) and immunohistochemical staining for CD30. Therefore, we devised a notation that we termed proportion of immunoreactivity/expression for FCM (PRIME-F notation), based on the cellular proportion showing different antigen-antibody reactivity.

**Methods:**

We retrospectively compiled 211 cases of T-cell lymphoma, assessed via FCM, from major hospitals in Miyagi Prefecture from January 2012 to January 2019, and compared 52 of these cases with the immunohistochemical immunoreactive (IR) pattern of CD30 (PRIME-I notation). The PRIME-F notation was divided into five levels: notations starting with “-” followed by 3, 2, and 1 “>” correspond to level-I, level-II, or level-III; notations starting with “(dim)+” correspond to level-IV; and those starting with “+” or “(bright)+” correspond to level-V.

**Results:**

The 52 cases of PRIME-F notation with “+” included 16 cases of peripheral T-cell lymphoma (PTCL/NOS), 3 of follicular T-cell lymphoma (FTL), 3 of angioimmunoblastic T-cell lymphoma (AITL), 6 of extranodal NK/T-cell lymphoma/nasal type (ENKL), 18 of adult T-cell lymphoma (ATL), and 6 cases of anaplastic large cell lymphoma (ALCL). Eight of the 52 cases were immunohistochemically CD30-negative. In the PRIME-F level-I to III group (excluding false-positive cases), 21.7% (5 out of 23 cases) were < 10% positive for CD30 upon immunohistochemistry (IHC). Contrarily, in the level-IV & -V group, no CD30 positivity rate of < 10% upon IHC was found (0%) (*p* = 0.0497). In level-IV, 42.9% of cases presented a CD30 negative rate > 1/3 upon IHC, while in level-V, only 7.1% (one out of 14 cases) did. The CD30 negative rate tended to be low (*p* = 0.0877) in level-V.

**Conclusions:**

To our knowledge, this is the first report describing the correspondence between FCM and immunohistochemistry findings for CD30 through newly proposed notations. The PRIME-F and PRIME-I notations for CD30 showed a minor positive correlation. The PRIME notation is considered universally applicable to antibodies, and notations of both FCM and IHC show great potential for big data.

**Supplementary Information:**

The online version contains supplementary material available at 10.1186/s12885-021-09098-4.

## Background

The immunological phenotype of leukemia is currently determined by flow cytometry (FCM); however, to diagnose malignant lymphoma, immunohistochemistry is performed along with FCM to examine the immunological phenotype [1, 2]. FCM has the following advantages such as i) rapidity of results, ii) semi-quantitative measurement of cell size and immunoreactive (IR) patterns, and iii) capability of multiparameter analysis [3]; however, its disadvantages include: i) the necessity of unfixed tissue, ii) indirect observation and sensitivity of detection of cells, and iii) primarily searching for cell surface antigens. FCM is, therefore, complemented by immunohistochemistry resulting in both being commonly used to diagnose malignant lymphoma.

Immunohistochemistry and histopathological findings are generally recorded by pathologists; however, FCM results are often displayed as a percentage of the positive rate, which can be represented by a bar graph or multiple two-dimensional plots [1]. FCM results are only illustrated with a panel of figures. Hence, it is difficult to integrate the findings of flow cytometry with those of histopathology, chromosome analysis, and genetic analysis. FCM findings are, therefore, rarely described in pathology reports. Even when described, the method of expressing the findings depends on the interpreter’s background, beliefs, aesthetic sense, ingenuity, personality, etc., and there is currently no established universal notation for expressing FCM findings. In flow cytometry, the numerical value of the positive rate and the bar graph, the dot distribution on the two-dimensional plots, i.e., the difference in fluorescence intensity derived from the intensity of different antigen-antibody reactions on the cell surface, and the composition ratio of each fluorescence intensity (cellular proportion showing different antigen-antibody reactivity, CPAR) cannot be analogized.

Brentuximab vedotin with cyclophosphamide, doxorubicin, and prednisone is the standard of care for patients with CD30-expressing peripheral T-cell lymphoma. It is important to confirm the CD30 expression in the lymphoma cells [4]. The relationship between the expression levels of CD30 and therapeutic efficacy is not clear; however, an expression as low as 1% exhibits effects, in both T-cell and B-cell lymphomas [5, 6]. Therefore, to determine the appropriateness of the anti-CD30 therapy, it is important to confirm the CD30 expression in the lymphoma cells, irrespective of the expression levels. In Japan, immunohistochemistry for CD30 is not routinely performed for all cases of lymphoma. In many cases, FCM is used to screen for CD30, and the T, B, and NK antigens, in the diagnosis of malignant lymphoma.

Therefore, in this study, we propose a method that allows CPAR to be understood through commonly used notation symbols in accordance with certain methods, rather than simply expressing the results of FCM as numerical values ​​or bar graphs. This study examines the utility of this method through comparison with immunohistochemical notation of CD30, which is now one of the companion diagnostics.

## Methods

### Case selection

We retrospectively compiled all T-cell lymphoma cases forwarded to the Registration-Examination-Analysis-Description (READ) system® (Life Science Institute, Tokyo, Japan) between January 2012 and January 2019 from major hospitals in Miyagi Prefecture, which were assessed via FCM with a sufficient number of cells (211 cases). Thereafter, 52 cases including the IR pattern of “+” through the evaluation of CD30 expression via FCM using PRIME-F notation (described later) were included.

### Diagnosis of malignant lymphoma

**Fig. 1:**
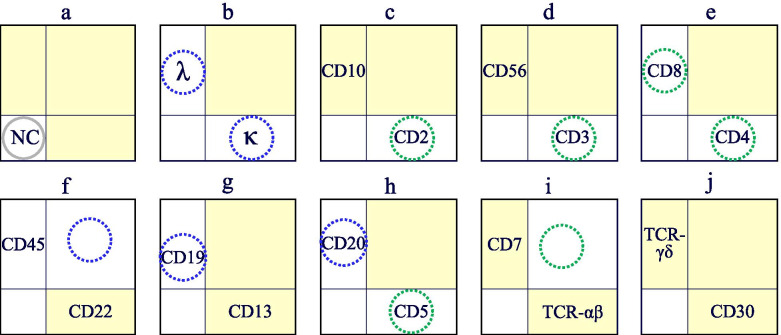
Panel showing flow cytometry results, comprising 10 two-dimensional histograms with yellow quadrants. NC: negative antibody control κ: immunoglobulin kappa λ: immunoglobulin lambda. Letters from “a” to “j” are assigned in the order of the two-dimensional histogram. Reactive B- or T lymphocyte groups are located in blue or green dotted circles, respectively. No cell groups were identified in the yellow quadrants. Therefore, if significant cell groups were detected during lymph node analysis, abnormal cell populations (ACPs) may be present, indicating the presence of neoplastic lesions

Malignant lymphoma was diagnosed by expert pathologists (hematopathologists) by morphological examination (hematoxylin and eosin-stained slides combined with immunohistochemistry) along with FCM (FACS Calibur HG, Cell Quest Pro, Becton, Dickinson and Company), cytogenetic (G-band method with or without fusion FISH), and molecular analyses (detection of IgH/T-cell receptor beta (*TCRβ*) genes via Southern blotting with or without PCR) based on the 2017 WHO classification [2]. We used two-color flow cytometry; the diagnosis was made using only tissue biopsy (or resection) specimens. The findings in the bone marrow, peripheral blood, and body fluids were not confirmed, at least at the time of diagnosis. And we used same specimens for FCM and IHC. The FCM results are shown in a panel comprising ten types of two-dimensional plots (Fig. 1), and the antibodies used in this study are shown in the respective two-dimensional plots. CD30 is indicated with a horizontal axis in j of the two-dimensional plots diagram, and TCRγδ is arranged along the vertical axis. Immunohistochemistry for CD30 was performed using the Ventana Benchmark Ultra automated immunostainer (Ventana Medical Systems (VMS), Tucson, AZ, USA) or the Bond-III Leica automated slide stainer. Appropriate positive and negative controls were included. The antibodies used in the FCM and the IHC were listed in the supplementary Table.

### FCM evaluation method

**Fig. 2:**
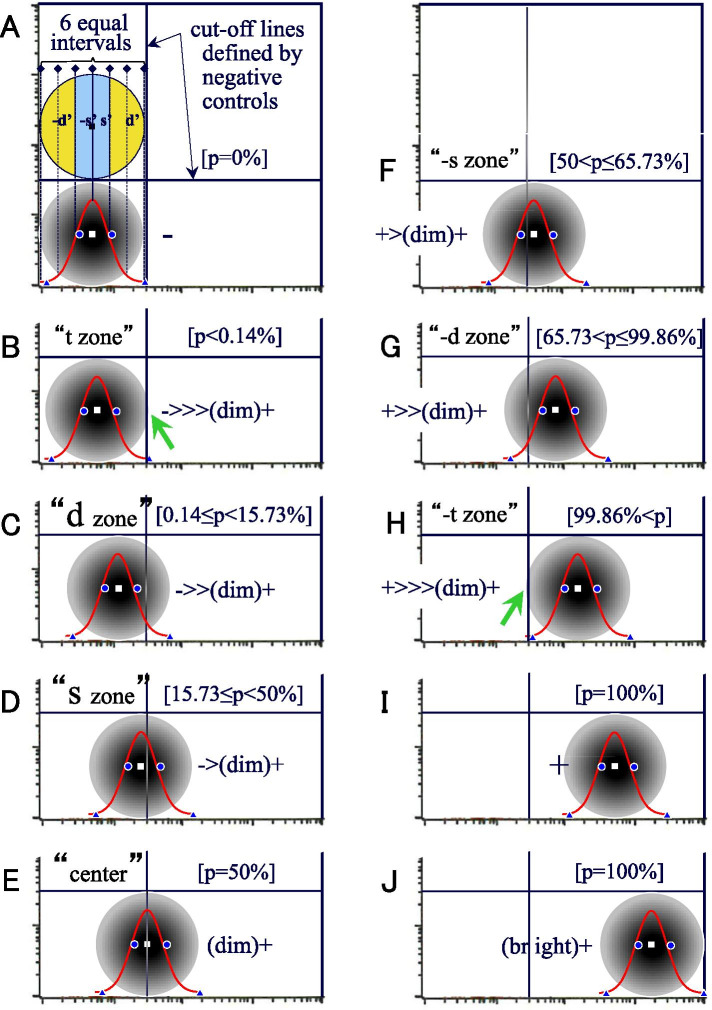
Two-dimensional histograms with circular two-dimensional dot-plots overlaid with a single histogram with a normal distribution. The aforementioned notation is shown in the right (or left) lower quadrant, and the positive rate (p) on the right side of the cutoff line is shown in square brackets in the right upper quadrant. If the center and edge of the circle are all to the left of the cutoff line, the expression is “-” (**A**), on the right side of the cutoff line “+” (**I**), significantly stronger than normal strength “(strong)+” (**J**), the center of the circle overlaps the cutoff line “(dim)+” (**E**). The center of the circle is on the negative (−) or positive (+) side of the cutoff line; thereafter, the degree of “dim” should be indicated with numerous inequality signs (1 to 3) depending on which zone the cutoff line is in (shown in the left upper quadrant from **B**, **C**, **D**, **F**, **G** and **H**)

Proportion of immunoreactivity/expression notation for FCM (PRIME-F notation) was devised as a method to express the CPAR of tumor cells. The outline of PRIME-F notation is as follows: “In a two-dimensional histogram, first the center of circular two-dimensional dot-plots were determined, and expressed using three symbols +, -, (dim) + and up to three “>” (Fig. 2). If the center and edge of the circle are all to the left of the cutoff line, the expression should be “-” (A); when located at the normal fluorescence intensity on the right side of the cutoff line, “+” (I); when it is significantly stronger than normal strength, “(strong)+” (J). If the center of the circle overlaps the cutoff line, the expression should be “(dim) + .” Otherwise, first it should be clarified whether the center of the circle is on the negative (−) or positive (+) side of the cutoff line; thereafter, the degree of “dim” should be indicated with numerous inequality signs (1 to 3) depending on which zone the cutoff line is in (shown in the left upper quadrant from B to J).

**Fig. 3:**
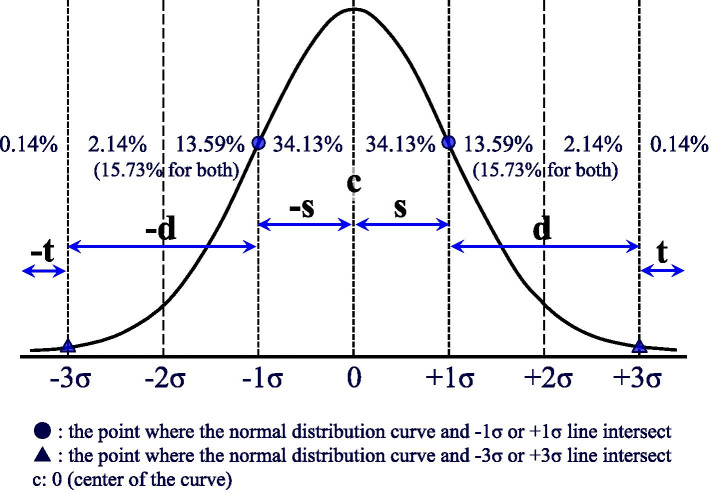
A normal distribution curve has been used to divide circular two-dimensional dot-plots into six zones. Normal distribution curve for circular two-dimensional dot-plots is divided into six zones as follows from the left: “-t zone” < −3σ, 3σ≦ “-d zone” < −1σ, −1σ≦ “-s zone” < 0, 0 < “s zone” ≦ + 1σ, +1σ < “d zone” ≦ + 3σ, 3σ < “t zone”

**Fig. 4:**
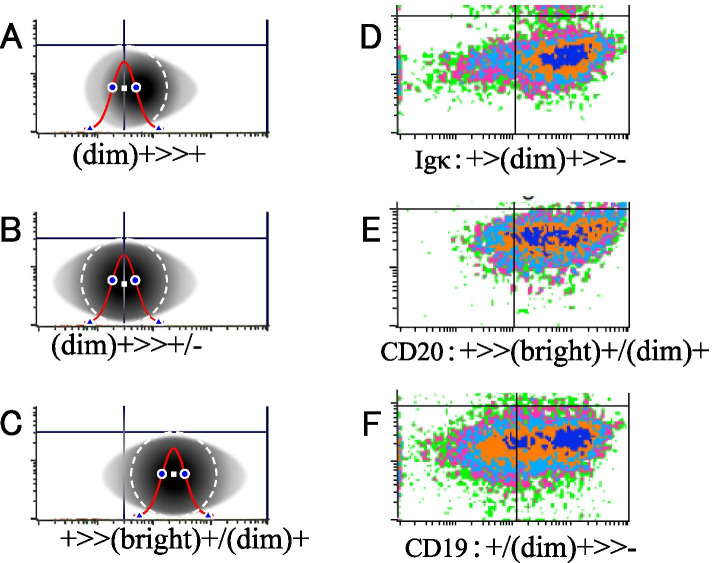
Two-dimensional histograms with non-circular two-dimensional dot distribution and density plots of cases with non-normal distribution. Two-dimensional histograms with non-circular distribution using a schematic representation (**A-C**), and that of actual cases using density plots **D-F**; Blue color represents the highest density of the events in the cell population, and the color changes in the order of orange, light blue, pink, and green as the density decreases. This indicates that the cell clusters identified using flow cytometry are not always round, because they do not always have a normal (Gaussian) distribution. **A-C** The semi-elliptical (**A**) and the elliptical distribution (**B**, **C**) are overlayed with the main, single circular histogram (white dotted circle). **D** The single highest density area (blue, corresponding to that around the vertex of the distribution) is almost cohesive; however the left and right base distributions of the whole cell population is considerably more uneven than that of **A** and are not normally distributed. **E** The center of the highest density area and the vertex of the distribution is not clear because the distribution of blue dots is disorganized and wide. **F** There are two high-density areas (larger and smaller), each of which appears to have an overlapping base, resulting in a wide distribution as a whole

The method of dividing the diameter was based on the basic concept of a normal distribution of -3σ, −1σ, 0, 1σ, and 3σ as the boundary to divide the circle into six zones: “-t zone,” “-d zone,” “-s zone,” “s zone,” “d zone,” and “t zone” (Fig. 3). However, since a normal distribution curve is not plotted on a two-dimensional plots chart, it is not practical. The 6-equal division method was substituted (Fig. 2A). Nevertheless, it is usually difficult to superimpose normal distribution curves in the flow panel or to express the p ratio with the exact values shown in the figure (“0.14%,” “15.73%,” “66.73%,” and “99.86%”). Therefore, the following simplified method was used. If circular two-dimensional dot-plots tangentially touch the cutoff line (= tangential contact), that contact should be substituted for a -t or t zone (green arrows in B and I). If circular two-dimensional dot-plots overlap deeper than the cutoff line and tangential contact, as shown in the left upper quadrant in A, the diameter of circular two-dimensional dot-plots should be divided into six equal parts from the left, 1/3, 1/6, 1/6, and 1/3 corresponding to the -d ‘zone, −s’ zone, s ‘zone, and d’ zone (d = dandelion color, s = sky-blue color), respectively. Although this is considered a simple method, there is almost no difference between the normal distribution curves -1σ and 1σ in the left lower quadrant of A. If the two-dimensional dot-plots are elliptical, the main circular dimensional dot-plots are defined at the center and the region extending from the center is expressed by adding “+, -, (dim)+ and (bright)+”. In addition, three cases that did not exhibit a normal distribution are also presented using density plots (BD Cell Quest Pro™ software of Becton Dickinson & Company Ltd.) (Fig. 4). The FCM data were analyzed in a blinded fashion by two expert hematopathologists, respectively.

### Evaluation method for immunohistochemistry

**Fig. 5:**
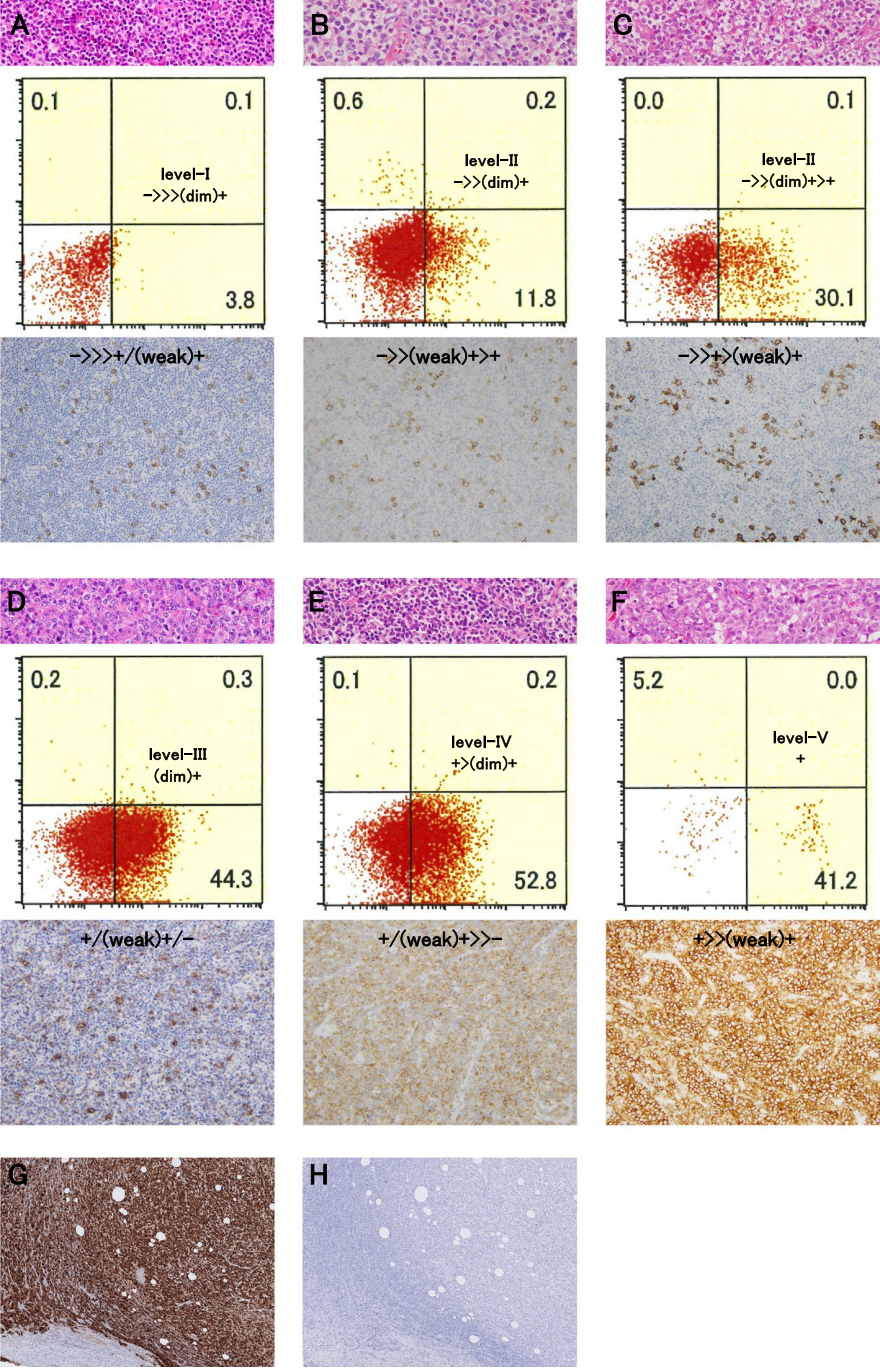
Various CD30 expression patterns determined via flow cytometry and immunohistochemistry. Examples of level-I (**A**) to V (**F**) notations of flow cytometry and hematoxylin & eosin stain (top), and flow cytometry and immunohistochemistry (bottom). Notations of flow cytometry and immunohistochemistry are distinguished in the figures. CD30-positive cases with negative control (**G**, **H**)

Not only the proportion of tumor cells positive for a specific antigen, but also the notation based on the proportion of immunoreactivity/expression for immunohistochemisty (PRIME-I) was used [7]. The evaluation was performed manually/ visually by two expert hematopathologists. Negative control for CD30 is included (Fig. 5).

### Statistical analysis

JMP Pro version 14.2.0 software (SAS Institute, Inc., Cary, NC, USA) was used for all statistical analyses. Associations and correlations between two variables were identified using the Fisher exact test. A *P* value   < 0.05 was considered statistically significant.

## Results

The classification of the 211 cases examined by FCM is shown in Table 1. In this study, 52 cases of PRIME-F notation with “+” included 16 cases of peripheral T-cell lymphoma/NOS, 3 of follicular T-cell lymphoma, 3 of angioimmunoblastic T-cell lymphoma, 6 of extranodal NK/T-cell lymphoma/nasal type, 18 of adult T-cell lymphoma, and 6 cases of anaplastic large cell lymphoma (Table 1). The PRIME-F notation was divided into five levels via the following method and compared with the immunohistochemical CD30 IR pattern expressed via PRIME-I notation at each level (Table 2). Notations starting with “-” followed by 3, 2, and 1 “>” are level-I, level-II, and level-III, respectively. Notations starting with “(dim)+” are level-IV, and those starting with “+” or “(bright)+” are level-V.

**Table 1:** T-cell lymphoma subtypes examined in this study by both flow cytometry and immunohistochemistry

Subclassification	Number of cases examined by flow cytometry	Number of cases positive for CD30 by flow cytometry (percentage to left column)	Number of cases positive for CD30 by immunohistochemistry (percentage to left column)
PTCL	104	22 (21.1%)	17 (77.2%)
AITL	39	3 (7.7%)	1 (33.3%)
ALT	35	18 (51.4%)	15 (83.3%)
ALCL	17	6 (35.3%)	6 (100%)
ENKL	16	6 (37.5%)	6 (100%)

Abbreviations: *PTCL* peripheral T-cell lymphoma, NOS, *AITL* angioimmunoblastic T-cell lymphoma, *ATL* adult T-cell lymphoma, *ALCL* anaplastic large cell lymphoma, *ENKL* extranodal NK/T-cell lymphoma/nasal type

Two hundred eleven T-cell lymphoma cases biopsied from 2012 to 2019 were examined by flow cytometry. Positivity for CD30 was not obtained by flow cytometry in the following subtypes; subcutaneous panniculitis-like T-cell lymphoma, mycosis fungoides, primary cutaneous CD30-positive lymphoproliferative disorder, and enteropathy-associated T-cell lymphoma

**Table 2:** Comparison of CD30 reactivity between flow cytometry by PRIME-F notation and immunohistochemistry by PRIME-I notation

	Subclassificatioin	Site	PRIME-F notation for FCM	Notation level	PRIME-I notation for IHC
1	AITL	LN	->> > (dim)+	I	–
2	AITL	tonsil	->> > (dim)+	I	–
3	ATL	LN	->> > (dim)+	I	–
4	ATL	LN	->> > (dim)+	I	–
5	ATL	LN	->> > (dim)+	I	–
6	ENKL	nasal cavity	->> > (dim)+	I	->> > (weak) + ^a^
7	PTCL	stomach	->> > (dim)+	I	->> > +/(weak) + ^a^
8	ENKL	nasal cavity	->> > (dim)+	I	-> > +/(weak)+
9	PTCL	skin	->> > (dim)+	I	-> > +/(weak)+
10	PTCL/FHT	LN	->> > (dim)+	I	-> > +
11	ENKL	nasal cavity	->> > (dim)+	I	- > (very weak)+> > (weak)+
12	ATL	LN	->> > (dim)+	I	+/(weak)+/−
13	ALCL	LN	->> > +	I	+
14	FTL	LN	-> > (dim)+	II	–
15	FTL	LN	-> > (dim)+	II	–
16	PTCL/FHT	LN	-> > (dim)+	II	–
17	ATL	LN	-> > (dim)+	II	->> > (very weak) + ^a^
18	ATL	LN	-> > (dim)+	II	->> > (weak) + ^a^
19	ENKL	nasal cavity	-> > (dim)+	II	-> > (weak)+
20	PTCL	LN	-> > (dim)+	II	-> > (weak)+ > +
21	PTCL	LN	-> > (dim)+	II	-> > +/(weak)+
22	ATL	LN	-> > (dim)+	II	- > (very weak)+
23	ENKL	LN	-> > (dim)+	II	(very weak)+/−
24	ATL	LN	-> > (dim)+	II	+/(weak)+/−
25	PTCL	LN	-> > (dim)+	II	(weak)+>/(very weak)+ > −
26	ATL	LN	-> > (dim)+ > +	II	->> > (weak) + ^a^
27	PTCL	LN	-> > (dim)+ > +	II	-> > + > (weak)+
28	PTCL	LN	-> > (dim)+ > +	II	-> > +/(weak)+
29	FTL	thyroid gland	- > (dim)+ > +	III	- > (weak)+
30	ENKL	nasal cavity	- > (dim)+	III	-> > (weak)+
31	ATL	LN	- > (dim)+	III	+/(weak)+/−
32	AITL	LN	(dim)+	IV	+/(weak)+/−
33	ATL	LN	(dim)+	IV	+/(weak)+/−
34	ATL	LN	(dim)+	IV	+/(weak)+> > −
35	ATL	skin	(dim)+	IV	+/(weak)+
36	PTCL	LN	(dim)+	IV	+
37	ALCL	testis	(dim)+	IV	+
38	PTCL	tonsil	(dim)+> > +	IV	- > (weak)+ > +
39	ALCL	LN	+/(dim)+/−	V	+> > (weak)+
40	ALCL	LN	+ > (dim)+ > −	V	+
41	PTCL	LN	+ > (dim)+	V	+/(weak)+> > −
42	ATL	LN	+>> > (dim)+	V	+/(weak)+/(very weak)+
43	PTCL	LN	+>> > (dim)+	V	+
44	ATL	LN	+> > (dim)+	V	+/(weak)+/−
45	ATL	skin	+> > (dim)+	V	+ > (weak)+> > (very weak)+
46	ATL	LN	+> > (dim)+	V	+
47	ALCL	LN	+	V	+>> > (weak)+
48	PTCL	LN	+	V	+> > (weak)+
49	ATL	LN	+	V	+
50	PTCL/γδ-T	pleural effusion	(bright)+>> > +/(dim)+	V	+
51	ALCL	bone	(bright)+	V	+ > (weak)+ > −
52	PTCL/γδ-T	skin	(bright)+	V	+

Abbreviations: *FCM* flow cytometry, *LN* lymph node, *FTL* follicular T-cell lymphoma, *FHT* follicular/helper phenotype, *PTCL* peripheral T-cell lymphoma/NOS, *AITL* angioimmunoblastic T-cell lymphoma, *ENKL* extranodal NK/T-cell lymphoma/nasal type, *ATL* adult T-cell lymphoma, *ALCL* anaplastic large cell lymphoma

^a^percentage of CD30-positive cells is less than 10%

PRIME-F notation level is classified as follows. I, II, III: notations starting with ‘-’ followed by 3, 2, 1 “>”, respectively. IV: notation starting with ‘(dim)+’. V: notations starting with ‘+’ or ‘(bright)+’

Figure 5 shows the histological characteristics of typical cases as well as the FCM and immunohistochemistry findings of CD30. Levels I, II, and III indicate a relatively robust difference in the IR pattern of immunohistochemistry for CD30 (Fig. 5A, B, C, and D). Alternatively, levels IV and V did not display a significant difference in the IR pattern of CD30 (Fig. 5E and F). However, no clear correlation was observed between PRIME-F and PRIME-I.

As shown in Table 2, of the 52 T-cell lymphomas slightly positive in PRIME-F regardless of the IR pattern, immunohistochemistry revealed negative findings for CD30 (false-positive) in eight cases (15.4%) including five cases at level-I, and three at level-II. No such false-positive results were obtained at level-III, level-IV, and level-V. The subtypes of eight false-positive cases were angioimmunoblastic T-cell lymphoma, adult T-cell lymphoma, and T-cell lymphoma with a follicular/helper phenotype. Among the six cases of anaplastic large cell lymphoma, four were positive for anaplastic lymphoma kinase (ALK), and no significant difference was observed in PRIME-F notation of CD30 with ALK-negative cases.

In the PRIME-F level-I to -III group (excluding eight cases with false-positive findings), 5 of 23 cases (21.7%) were < 10% positive upon immunohistochemistry for CD30. On the other hand, none of the 21 cases in the level-IV & -V group had a CD30 positivity rate of < 10% upon immunohistochemistry (0%), which was significantly lower than the percentage in the level-I to -III group (*p* = 0.0497) (Table 3). Furthermore, although no significant difference was observed in terms of the positive rate between level-IV and level-V, in cases where the CD30 negative rate was > 1/3 upon immunohistochemistry, three out of seven cases (42.9%) were at level-IV, while only one of 14 cases (7.1%) was at level-V. Such a CD30 negative rate tended to be low (*p* = 0.0877) at level-V (Table 4). No significant differences were observed between level-I, level-II, and level-III.

**Table 3:** Comparison of CD30 positive rate obtained by immunohistochemistry between PRIME-F notation level-I-III and IV-V

	Cases with < 10% CD30 positive rate by immunohistochemistry^a^	Cases with > 10% CD30 positive rate by immunohistochemistry	Total
PRIME-F notation level-I-III	5	18	23
PRIME-F notation level-IV and V	0	21	21
total	5	39	44

Abbreviations: *PRIME-F* proportion of immunoreactivity/expression for flow cytometry

^a^Exclude the cases with CD30 negative by immunohistochemistry

**Table 4:** Comparison of CD30 negative rate obtained by immunohistochemistry between PRIME-F notation level-IV and V

	Cases with > 1/3 CD30 negative rate by immunohistochemistry	Cases with < 1/3 CD30 negative rate by immunohistochemistry	Total
PRIME-F notation level-IV	3	4	7
PRIME-F notation level-V	1	13	14
total	4	17	21

Abbreviations: *PRIME-F* proportion of immunoreactivity/expression for flow cytometry

## Discussion

We believe that the PRIME-F notation for FCM proposed in this study can semi-quantitatively represent CPAR in FCM, especially by comparing it with the PRIME-I notation for immunohistochemistry. Indeed, the notation of the PRIME-F method may be as complicated as that of PRIME-I, even when it is simplified by substituting the six equalities method. Nonetheless, such an impression of complexity may be attributed to the lack of efforts toward indicating FCM or immunohistochemistry findings in a systematic and consistent manner. Hence, it is important to abandon the use of the conventional notation and adopt this new notation.

The range of each zone of -d ‘, −s’, s ‘, d’, in the PRIME-F notation through the 6-division method is a zone generated by dividing the diameter including the -t zone and t zone into six equal parts. The ratio of the -t zone and t zone is only 0.28% when both are combined (Fig. 3). Therefore, there is almost no error from the range of -d, −s, s, and d zones in the normal distribution. Furthermore, although the cut-off line is drawn based on the negative control, it is drawn arbitrarily to some extent, and the error that may occur in the cutoff line itself is larger. As the positivity rate, which is widely used, is determined with the same cutoff line, the error due to the 6-segment method is no longer an issue. Moreover, as with immunohistochemistry, the basis of evaluation via the PRIME-F notation is not an accurate measure, but is used “to express the outline of CPAR by visual semi-quantitative evaluation”. Therefore, a certain level of width is inevitably generated during the evaluation of the positional relationship with the cutoff line. It is known that the positivity rate on the basis of a visual cutoff correlates well with the positivity rate determined by computerized image analysis in immunohistochemistry [8], and visual inspection is not only a sensory entity, but also a method of practical evaluation. Therefore, although the cutoff line has a somewhat ambiguous meaning, it can be assumed that visual inspection can compensate for this ambiguity to some extent. The irregular shapes of the dot distribution (Fig. 4D-F) limits its accuracy. However, the PRIME notation can express the general tendency of the distribution with a certain extent of accuracy.

In this study, two significant findings were obtained through visualization. First, in the PRIME-F level-I to -III group, the proportion of cases with a CD30 positivity rate of < 10% in PRIME-I was significantly lower than that in the level-IV & -V group, and CD30 positivity rates were > 10% in all cases upon immunohistochemistry in the level-IV & -V group. Therefore, it was shown that the level-IV & V group displayed a higher degree of positivity than the level-I to -III group. Furthermore, at level-V, the proportion of patients assessed via the PRIME-I notation and with a CD30 negativity rate of approximately one-third or greater tended to be lower than that of level-IV. Level-V displayed a relatively higher degree of CD30 immunoreactivity than level-IV. Consequently, although the PRIME-F and PRIME-I notations are not necessarily a one-to-one correspondence, the degree of notation can be considered parallel to some extent overall, indicating that visual semi-quantitative notation was significant and important during evaluation via FCM and immunohistochemistry. Furthermore, their notations of both FCM and immunohistochemistry show great potential for big data.

In the READ system currently in use, the actual pathology report includes, for example, “Flow cytometry recognizes the existence of abnormal B cell population indicating CD45+ CD10->(dim)+ CD19(dim)+>>+/- CD20(bright)+ CD22+>>(dim)+ kappa+>>(dim)+ lambda- (clear light chain restriction) CD2- CD3- CD5- CD7- TCRαβ- TCRγδ- CD4- CD8- CD56- CD13- CD30-”. We developed this notation such that the positional relationship between two-dimensional dot-plots and cutoff lines of each antigen can be easily inferred from the contents. Thus, FCM findings can be easily integrated with other results including histopathology, chromosome analysis, and genetic analysis. Furthermore, FCM results can be pathologically recorded as a batch. Moreover, without considering the two-dimensional plots panel, it would be possible to compare the CPAR of the PRIME-F notation at onset and relapse. Similarly, for the PRIME-I notation, even with no remaining immunohistochemical specimen, the IR pattern of the case can be inferred using the previously described PRIME-I notation across time and space.

A previously described method can be used to determine positive or negative percentages in the FCM results with cutoff values of 10% [9] to 20% [10]. These (numerical values ​​of 10 to 20%) are the original cutoff values ​​to diagnose acute leukemia, and it is difficult to determine whether they reflect a truly positive image. However, thereafter, other than leukemia phenotyping, the 20% cutoff has been used for positive and negative values [11] and is also currently applicable [12]. However, in this study, even if it is ->> > (dim) + (level-I) and -> > (dim) + (level-II), which are equivalent to less than 20% in FCM, up to 50 and 70% CD30-positive tumor cells have been detected, respectively (Fig. 5, A and B) via immunohistochemistry. Therefore, if the positivity rate is determined with a cutoff value of 20% through FCM, such a case would be overlooked. Hence, a cutoff of 20% is meaningless. Regardless of the IR pattern, if the PRIME-F notation is determined to be level-I or level-II corresponding to a positive rate of < 20%, immunohistochemistry for CD30 should be performed along with FCM. However, in angioimmunoblastic T-cell lymphoma, sometimes activated B lymphocytes may be visualized as CD30-positive through FCM; hence, a comprehensive decision is required by combining cellular imaging and immunohistochemistry for T antigens.

Studies have reported that among all subtypes of T-cell lymphoma, approximately 60% of cases display CD30 positivity upon immunohistochemistry [4]. However, in this study, 52 cases (24.6%) out of 211 cases were suspected as CD30-positive through FCM alone because CD30-positive cells tend to be larger; therefore, there was low sensitivity due to low cell count and it was difficult to detect CD30 positivity in the cytoplasm via FCM. Moreover, while the diagnostic criterion for anaplastic large cell lymphoma is CD30 positivity, only 6 (35.3%) of 17 cases have been confirmed through flow cytometry. In recent years, numerous CD30-positive cells have been detected in extranodal NK/T-cell lymphoma/nasal type [4, 13, 14]; however, few cases were considered here, probably because FCM could not be carried out with a sufficient number of cells owing to a high tendency for tumor necrosis. Furthermore, angioimmunoblastic T-cell lymphoma and adult T-cell lymphoma are prone to false-positive findings and should be interpreted with care [4, 12]. Accordingly, the detection rate of CD30 via FCM alone was lower than that through immunohistochemistry, and false positives could occur occasionally; however, to take advantage of factors, such as the rapidity of FCM results, semi-quantitative IR pattern, and an index for performing immunohistochemistry, the use of FCM in determining the immunological phenotype of malignant lymphoma is important for evaluating CD30 expression.

One of the limitations of this study is that the same laboratory and equipment were used for FCM in all cases, but it will be difficult to completely standardize the results if the study is conducted at multiple facilities and with multiple equipment in the future. In addition, it is difficult to simply compare the results of FCM and IHC in some aspects. On the contrary, the number of facilities using automatic immunostaining device has been increasing recently, and to some extent, differences between facilities are decreasing, leading to the spread of companion diagnostics.

Finally, the PRIME-F and PRIME-I notations unify the findings of FCM and immunohistochemistry when conducting an epidemiological study involving annual and permanent prognostic follow-up of patients with lymphoid tumors in Miyagi Prefecture, implemented by the “Ichinohasama Memorial READ Blood Academy” established in October 2008 (Miyagi study) [15, 16].

## Conclusions

This study is the first to express CPARs with known symbols based on original premises and to compare FCM with immunohistochemistry. The PRIME notation has potential for universal applicability to antibodies other than the anti-CD30 antibody used in this study, and multivariate analysis of FCM and immunohistochemistry big data in malignant lymphoma has the potential to show the relationship between genetic mutations and translocations, tumor malignancy, and prognosis.

## Supplementary Information

**Additional file 1.**

## Data Availability

The datasets used and/or analyzed during the current study are available from the corresponding author on reasonable request.

## Abbreviations

FCM: Flow cytometry
IR: Immunoreactive
IHC: Immunohistochemistry
PRIME-F: Proportion of immunoreactivity/expression for flow cytometry
PRIME-I: Proportion of immunoreactivity/expression for immunohistochemistry
CPAR: Cellular proportion showing different antigen-antibody reactivity
READ: Registration-Examination-Analysis-Description
IGH: Immunoglobulin heavy chain
TCR: T-cell receptor
FISH: Fluorescence in situ hybridization
FTL: Follicular T-cell lymphoma
PTCL: Peripheral T-cell lymphoma, NOS
AITL: Angioimmunoblastic T-cell lymphoma
ATL: Adult T-cell lymphoma
ALCL: Anaplastic large cell lymphoma
ENKL: Extranodal NK/T-cell lymphoma/nasal type
